# Leaf–air temperature difference as a reliable indicator for potato water status

**DOI:** 10.3389/fpls.2025.1609350

**Published:** 2025-09-01

**Authors:** Peng Liu, Ting Guan, Mingshou Fan, Jiawei Guo, Meirong Wang, Zhihui Shang, Liguo Jia

**Affiliations:** College of Agronomy, Inner Mongolia Agricultural University, Hohhot, China

**Keywords:** water stress, infrared thermometry, moisture diagnosis, leaf position, precision irrigation

## Abstract

**Introduction:**

Potato (*Solanum tuberosum L*.) production in semi-arid regions requires precision irrigation management to address water scarcity, highlighting the critical need for real-time, non-destructive plant water status assessment techniques. This study aimed to investigate the feasibility of measuring the leaf–air temperature difference (LAD) as an indicator for diagnosing potato water status.

**Methods:**

A field experiment was conducted with five irrigation levels (0–300 mm) to evaluate LAD responses at three leaf positions (L_1_, L_4_, and L_8_) across different growth stages.

**Results:**

The results demonstrated that LAD significantly correlated with irrigation levels, plant water content (PWC), and soil moisture, with the strongest relationships observed for the fourth leaf from the top (L_4_). L_4_ exhibited the highest sensitivity to water status, the lowest variability among plants. A binomial regression between LAD and yield was identified, revealing a threshold LAD beyond which further LAD increases did not enhance the yield. These findings not only suggest that LAD can be a reliable indicator for monitoring potato water status but also identify L_4_ as the optimal leaf position for LAD-based water status monitoring.

**Discussion:**

The study provides a foundation for precision irrigation in potato production, enabling improved water use efficiency and sustainable potato production in a semiarid region.

## 1 Introduction

Potato (*Solanum tuberosum L.*) has a large water requirement. Approximately 100–150 L of water is required to produce 1 kg of fresh tubers (Men and Chen, 1986; Hill et al., 2021). In semi-arid regions such as Yin Mountain area, a dominant potato production zone in China (Yang et al., 2022), precipitation often fails to meet the water requirements of potato cultivation, making irrigation essential for optimal yields. However, water resources are often scarce in these regions. Therefore, implementing efficient irrigation management strategies to concurrently enhance potato yield and water use efficiency is crucial for the sustainable development of the potato industry in the regions (Zhao et al., 2016, 2018; Akkamis and Caliskan, 2023). This challenge underscores the need for non-destructive, real-time monitoring techniques to assess plant water status—a foundation of precision irrigation (Ihuoma and Madramootoo, 2017).

Plant transpiration plays a critical role in leaf temperature regulation, as water loss through stomata dissipates heat. Under water deficit conditions, reduced transpiration leads to increased leaf temperature, while adequate hydration maintains a lower leaf temperature (Gates, 1968). Handheld infrared thermometers, with their high accuracy and low noise levels, offer a promising tool for quantifying crop water stress through leaf–air temperature difference (LAD) measurements (Morales-Santos and Nolz, 2023; Dominic et al., 2024). Previous research in wheat (*Triticum aestivum L.*), maize (*Zea mays L.*), rice (*Oryza sativa L.*), sorghum (*Sorghum bicolor L.*), and other species had shown that the LAD could indicate well the plant water or soil water content (Zhang et al., 2017; Luan et al., 2021; Zhang et al., 2023; Kumari et al., 2024). In the meantime, temperature-based indices for irrigation such as the stress cumulative temperature and crop water stress index were established (Hiler and Clark, 1971; Idso et al., 1977; Jackson et al., 1977; Gardner et al., 1981; Idso et al., 1981; Clawson and Blad, 1982). These indices have been successfully applied in irrigation decision-making for crop production in many regions.

Despite these advances, the application of LAD for potato water status diagnosis remains uninvestigated. This knowledge gap stems from the complexity of potato’s pinnately compound leaves. Unlike gramineous and leguminous crops, potato leaflets in compound leaf exhibit age-dependent variations in flatness and wrinkling. These morphological variations pose challenges for obtaining consistent temperature measurements. Notably, the fourth leaf from the top of potato plants, as the first fully expanded leaf, exhibits relatively stable size and shape (Fan, 2019). Therefore, we hypothesize that this specific leaf may serve as an optimal site for diagnosing the crop’s water characteristics through LAD measurements. The objectives of this study were as follows: (1) to compare the LAD responses of different leaves to varying soil and plant water status and (2) to assess the feasibility of using a handheld differential infrared thermometer to measure LAD for real-time determination of potato plant water status.

## 2 Materials and methods

### 2.1 Description of the experimental area

**Figure 1:**
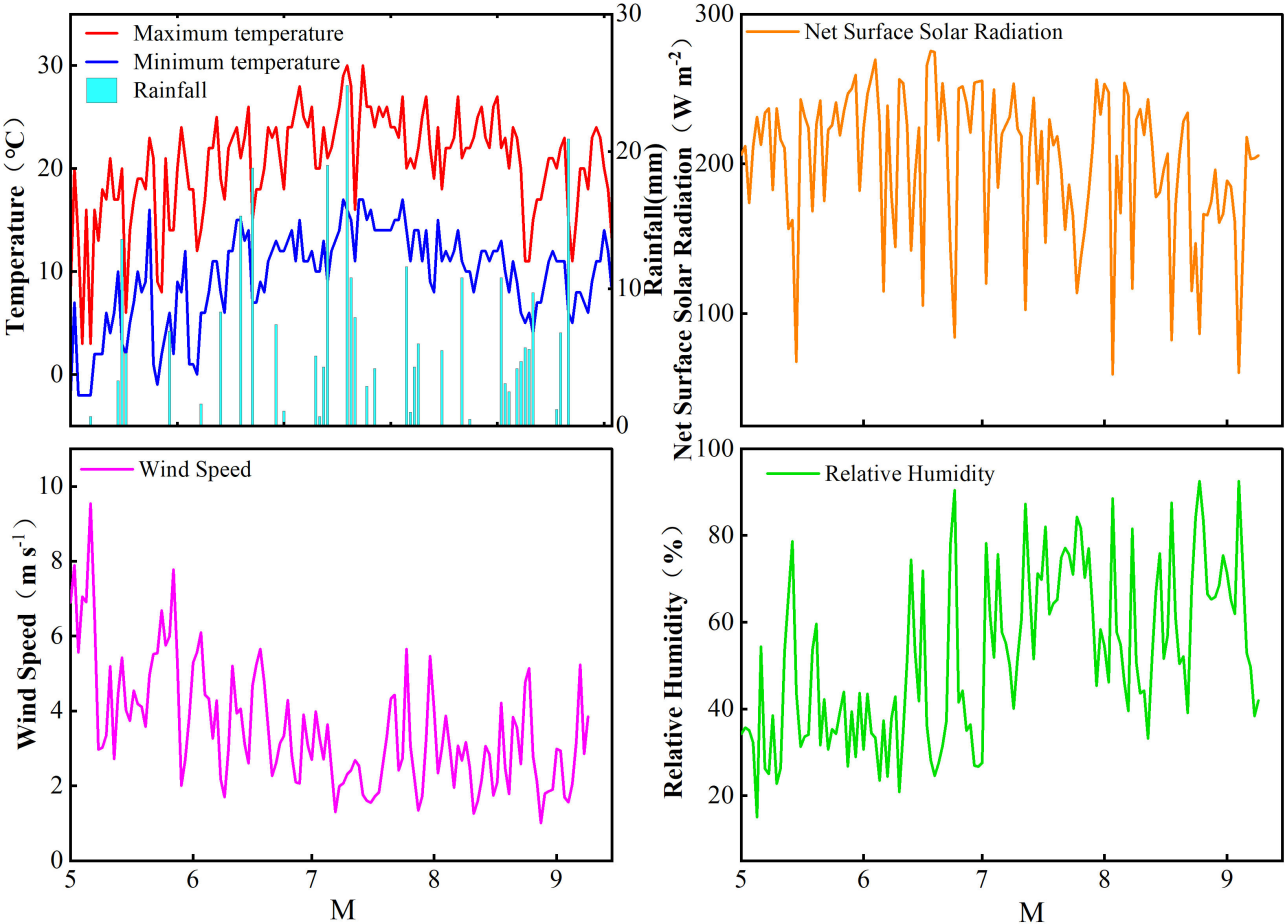
Maximum temperature, minimum temperature, rainfall, relative humidity, wind speed, and solar radiation during the potato growth period were recorded at the test site in 2021.

The field experiment was conducted from May to September 2021 in Chayouzhong County, Yinshan Mountain area, China (41°30′ N, 112°64′ E), which has a temperate continental monsoon climate, an average annual temperature of 1.3 °C, an elevation of 1,780 m, a frost-free period of approximately 100 days, and annual evapotranspiration greater than 2,000 mm. The soil at the experimental sites is sandy-loamy, with 26.3 g kg^-1^ of organic matter, 2.24 g kg^-1^ of total nitrogen, 10.3 mg kg^-1^ of available P (Olsen-P), 69.8 mg kg^-1^ of exchangeable K, pH of 7.9, and maximum field water holding capacity of 25.61%. The effective precipitation during the potato growth period was 236.6 mm. A small automatic weather station (Spectrum Watchdog 2900ET, USA) was installed in the experimental field to continuously and automatically detect rainfall, wind speed, air temperature, and other meteorological parameters during the potato growth period. The specific meteorological data are presented in **Figure 1**.

### 2.2 Experimental design

Five irrigation levels (total irrigation amount during potato growth), namely, 0 mm (W_0_), 75 mm (W_1_), 150 mm (W_2_), 225 mm (W_3_), and 300 mm (W_4_), were used in this experiment, and the specific irrigation regime (irrigation timing and amount of each irrigation event) for each treatment is shown in **Table 1**.

**Table 1:** Irrigation regime used in the experiment.

Date (month-day)	Irrigation amount (mm)				
	W_0_	W_1_	W_2_	W_3_	W_4_
06-28	0	7.5	15	22.5	30
07-04	0	7.5	15	22.5	30
07-10	0	7.5	15	25.5	36
07-17	0	7.5	22.5	33.8	45
07-24	0	13.5	23.4	33.8	45
07-31	0	13.5	23.4	33.8	45
08-10	0	13.5	23.4	33.8	45
08-20	0	4.5	12.3	19.5	24

W0 to W4 represent the total irrigation amounts applied during the entire growing season (0, 75, 150, 225, and 300 mm, respectively). The values in the table were allocated proportionally according to irrigation schedule.

### 2.3 Plant materials and crop management

The virus-free seed potato cultivar Kexin No.1 was used in the experiment. The plants were sown in May 2 and harvested in September 9. Drip irrigation was provided with a 0.3-m dripper spacing, and 2.2 L/h dripper flow was used. All of the treatments were arranged in a randomized block design with three replicates. The plot area was 90 m^2^, with 30 cm between potato plants and 90 cm between rows. Urea (300 kg N ha^−1^) was used as N source, and 30% of it was basal dressed and the 70% was fertigated through drip irrigation. Phosphorus fertilizer (79 kg P ha^−1^) and potassium fertilizer (224 kg K ha^−1^) were both broadcasted as basal fertilizers.

### 2.4 Measurements and methods

#### 2.4.1 Leaf–air temperature difference

A total of 12 potato plants were randomly selected from each plot at 35 days after emergence (DAE, tuber initiation stage), 50 DAE, and 60 DAE (tuber bulking stage) to measure air and leaf temperatures. The first (L_1_), fourth (L_4_), and eighth (L_8_) leaves from the top of the seedlings were selected as representative leaves of the upper, middle, and lower positions, respectively. A handheld differential infrared thermometer (AGRI-THERM III™, 6110 L, USA) was chosen for LAD detection in the experiment. The air temperature (T_a_) was measured at a distance of 10 cm perpendicular to the top leaflet at 13:00–15:00 for all treatments. The shady side of the leaflet was selected for measurement leaf temperature (T_i_). The air–leaf temperature difference (LAD) was calculated as follows:


$$\text{LAD}={\text{T}}_{a}-{\text{T}}_{i}$$

where T_a_ is the atmospheric temperature (°C), and T_i_ is the leaf temperature (°C).

#### 2.4.2 Plant biomass measurement

Five potato plants were randomly selected from each plot at 15, 35, 50, 60, and 80 DAE, and the plants were separated into roots, stems, leaves, and tubers. After fresh weight (FW) was taken, each part was placed in an oven at 105 °C for 30 min and then dried at 80 °C for 48 h to a constant weight. After the dried samples were weighed, this was recorded as dry weight (DW).


$$Dry\;matter\;weight\;\left({\text{g plant}}^{-1}\right)=\text{DW}.$$

Plant water content (%)=(FW−DW)/FW*100%.

#### 2.4.3 Leaf area index

At each sampling time, five plants in each plot were randomly sampled, and then the leaves from each plant were picked off. A small round leaf sample with a diameter of 1 cm from each leaf of 10 randomly selected leaves was taken using a puncher with a diameter of 1 cm. After drying, the dry weight of leaves per unit leaf area was then calculated accordingly. Based on the dry weight of the leaves of a single plant, the total leaf area of each potato plant was then derived. The leaf area index (LAI) was determined as the leaf area per unit field area.

#### 2.4.4 Leaf relative water content

After the fresh weight (FW) of the removed leaves was measured, the samples were immersed in water and allowed to fully absorb. The leaves were wiped dry and weighed again. This process was repeated until the weight became consistent, and the final result was recorded as the saturated fresh weight (SFW). The saturated water-absorbing leaves were placed in an oven at 105°C for 30 min, and then dried at 80 °C for 48 h to a constant weight, and the resulting weight was recorded as the dry weight (DW). The leaf relative water content (%) was calculated as follows:


LRWC=(FW−DW)/(SFW−DW)*100%

#### 2.4.5 Soil moisture

Soil moisture determination was performed on the same day as the LAD measurement. Soil samples from the 0–40-cm layers were taken directly by an auger at the midpoint between potato seedlings. Three replications were conducted for each treatment. After weighing, the soil samples were dried in an oven at 80 °C to a constant weight. The soil moisture was expressed as the percentage of water weight relative to the fresh weight.

#### 2.4.6 Tuber yield

At the end of the experiment, four 1.8-m^2^ area of normally growing potato plants from each plot were harvested for yield measurement, and a single tuber with a weight greater than 150 g was regarded as a commodity potato. The yield per hectare was converted according to the sampled yield.


$$\text{Percentage of commodity potatoes }\left(\%\right)={\text{Y}}_{M}/\text{Y }100\%$$

where YM represents the yield of tubers weighing ≥150 g, and Y denotes the total tuber yield.

### 2.5 Statistical analysis

The SPSS 25.0 statistical software package was employed to assess the normality of these data and conduct tests for homogeneity of variance. ANOVA with LSD test (*P* < 0.05) and regression analysis were performed, Pearson correlation coefficients were calculated to assess linear associations between normally distributed variables.

## 3 Results

### 3.1 Plant growth and tuber yield of potatoes under varying irrigation levels

**Figure 2:**
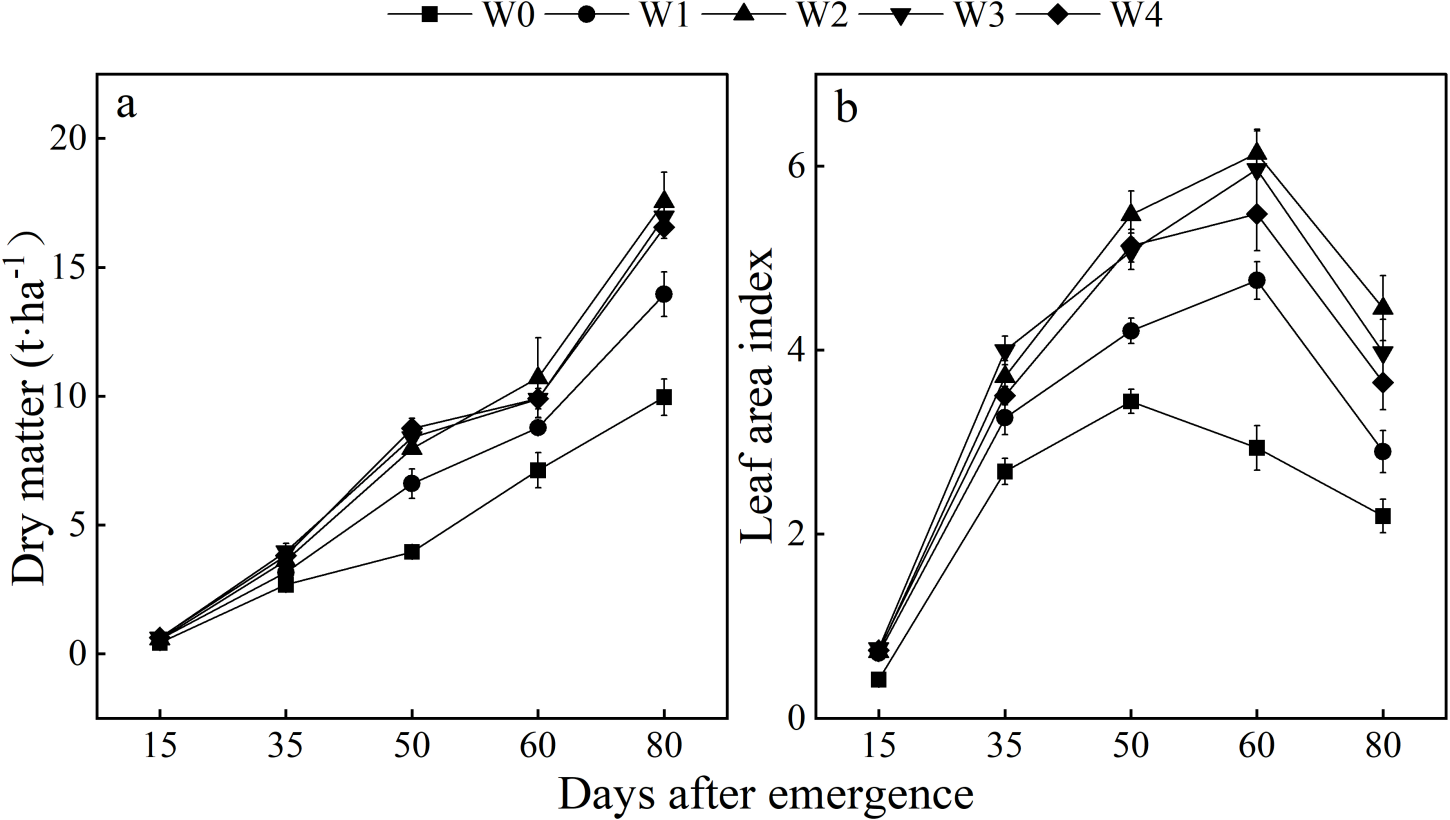
Plant dry matter and LAI of potatoes under different irrigation levels. **(a)** dry matter; **(b)** LAI. W_0_ (0 mm), W_1_ (75 mm), W_2_ (150 mm), W_3_ (225 mm), W_4_ (300 mm).

At the early growth stage, the plant dry weights did not show a significant difference among irrigation levels. Afterwards, both plant dry weight and leaf area index (LAI) of potatoes exhibited significant increases in response to higher irrigation rates (*p* < 0.05) (**Figure 2**).

Potato yield and its contributing factors demonstrated a remarkable response to irrigation under low-irrigation conditions (from W_0_ to W_2_). As the irrigation level increased within this range, there was an increase in tuber yield, the percentage of commodity potatoes, tuber number per plant, and the weight of tubers per plant. However, once the irrigation level surpassed 150 mm (W_2_), no further increases were observed in either the yield or its contributing factors, as shown in **Table 2**.

**Table 2:** Effects of varying irrigation levels on potato yield and yield components.

Treatment	Yield (t ha^-1^)	Commodity potato percentage (%)	Tuber number per plant	Tuber weight per plant (kg plant^-1^)
W_0_	27.98 ± 0.11 c	34.25 ± 1.85 c	5.18 ± 0.04 c	0.58 ± 0.06 c
W_1_	43.78 ± 0.49 b	65.92 ± 1.56 b	5.76 ± 0.13 b	0.88 ± 0.06 b
W_2_	54.71 ± 0.52 a	73.75 ± 1.21 a	6.98 ± 0.23 a	1.05 ± 0.04 a
W_3_	53.55 ± 0.36 a	72.95 ± 1.45 a	6.77 ± 0.19 a	1.06 ± 0.04 a
W_4_	53.46 ± 0.25 a	71.20 ± 0.36 a	6.71 ± 0.23 a	1.05 ± 0.04 a

Data are expressed as mean ± SE (*n* = 4). Means within a column followed by different lowercase letters differ significantly (*p* < 0.05) by LSD test after ANOVA. Irrigation treatments: total seasonal amounts of W_0_ (0 mm), W_1_ (75 mm), W_2_ (150 mm), W_3_ (225 mm), W_4_ (300 mm). Commercial potato percentage: tubers ≥150 g as proportion of total yield.

### 3.2 Leaf–air temperature difference influenced by irrigation levels

The irrigation level significantly affected LAD across all measured leaves (L1, L4, and L8). Compared to the non-irrigated treatment (W0), all irrigated treatments (W1–W4) resulted in higher LAD for each leaf. With increasing irrigation levels, the LAD of each leaf showed varying degrees of increase depending on the growth stage—for instance at 35 DAE, the LAD of L_4_ under W_4_ was 15% higher than that under W_1_. By 60 DAE, the LAD under W_4_ had increased by 38% compared to that under W_1_ (**Table 3**).

**Table 3:** Leaf–air temperature differences (°C) (LAD) under different irrigation levels.

Treatment	L_1_				L_4_				L_8_			
	35 DAE	50 DAE	60 DAE	Mean	35 DAE	50 DAE	60 DAE	Mean	35 DAE	50 DAE	60 DAE	Mean
W_0_	4.3	3.4	1.8	3.2c	4.2	3.9	2.8	3.7d	4.7	5.9	2.9	4.5d
W_1_	4.7	3.8	2.9	3.8b	5.2	5.3	5.5	5.3c	4.9	6.3	5.9	5.7c
W_2_	5.2	4.1	3.4	4.2ab	5.4	6.3	6.5	6.1b	5.4	6.8	7.9	6.7b
W_3_	5.3	4.2	4.3	4.6a	5.8	6.6	7.6	6.7a	6	7	8.3	7.1ab
W_4_	5.3	4.4	3.8	4.5a	6	7.2	7.6	6.9a	5.9	8	8.8	7.6a
Mean	4.9a	4.0b	3.2c		5.3b	5.9a	6.0a		5.4b	6.8a	6.8a	
*F* test												
Irrigation level (I)	9.35**				53.51**				26.74**			
DAE	33.98**				7.33**				18.68**			
I * DAE	0.97				4.66				6.56			

W_0_ (0 mm), W_1_ (75 mm), W_2_ (150 mm), W_3_ (225 mm), and W_4_ (300 mm). Values marked with different letters are significantly different at the 0.05 level (*p* < 0.05). L_1_ is the top leaflet of the first compound leaf from the top of the plant. L_4_ and L_8_ are the top leaflets of the fourth and eighth compound leaves from the top, respectively. *F*-values followed * or ** indicate the corresponding factor significantly affecting the leaf–air temperature differences at the level of *P*_0.05_ or *P*_0.01_. LSD method (*p* < 0.05) was used for the multicomparisons.

Statistical analysis indicated a significant positive correlation between the LAD and the irrigation level. At each growth stage, the correlation between the LAD of L_4_ or L_8_ and irrigation level reached a significance level of *p* < 0.01. Moreover, the correlation coefficient (R) between the LAD and irrigation level was greater for L_4_ than for L_1_ or L_8_ (**Table 4**).

**Table 4:** Correlation coefficient between the leaf–air temperature difference and irrigation amount on different days after emergence.

Days after emergence (DAE)	Leaf position		
	L_1_	L_4_	L_8_
35	0.226*	0.551**	0.329**
50	0.253*	0.479**	0.338**
60	0.619**	0.790**	0.764**

L_1_ is the top leaflet of the first compound leaf from the top of the plant. L_4_ and L_8_ are the top leaflets of the fourth and eighth compound leaves from the top, respectively. Values are Pearson correlation coefficients. * and ** represent significance at *p* < 0.05 and *p* < 0.01, respectively.​

Under the same irrigation level, the LADs of L_1_ exhibited the greatest variation among plants. The variation coefficient of LAD throughout the growth period was L_4_ < L_8_ < L_1_ (**Table 5**).

**Table 5:** Variation in leaf–air temperature difference.

Leaf position	Number of samples	Mean (°C)	Standard deviation (°C)	Coefficient of variation (CV) (%)
L_1_	600	4.6	1.69	36.70
L_4_	600	5.8	1.67	28.80
L_8_	600	6.0	1.96	32.70

L_1_ is the top leaflet of the first compound leaf from the top of the plant. L_4_ and L_8_ are the top leaflets of the fourth and eighth compound leaves from the top, respectively.

**Figure 3:**
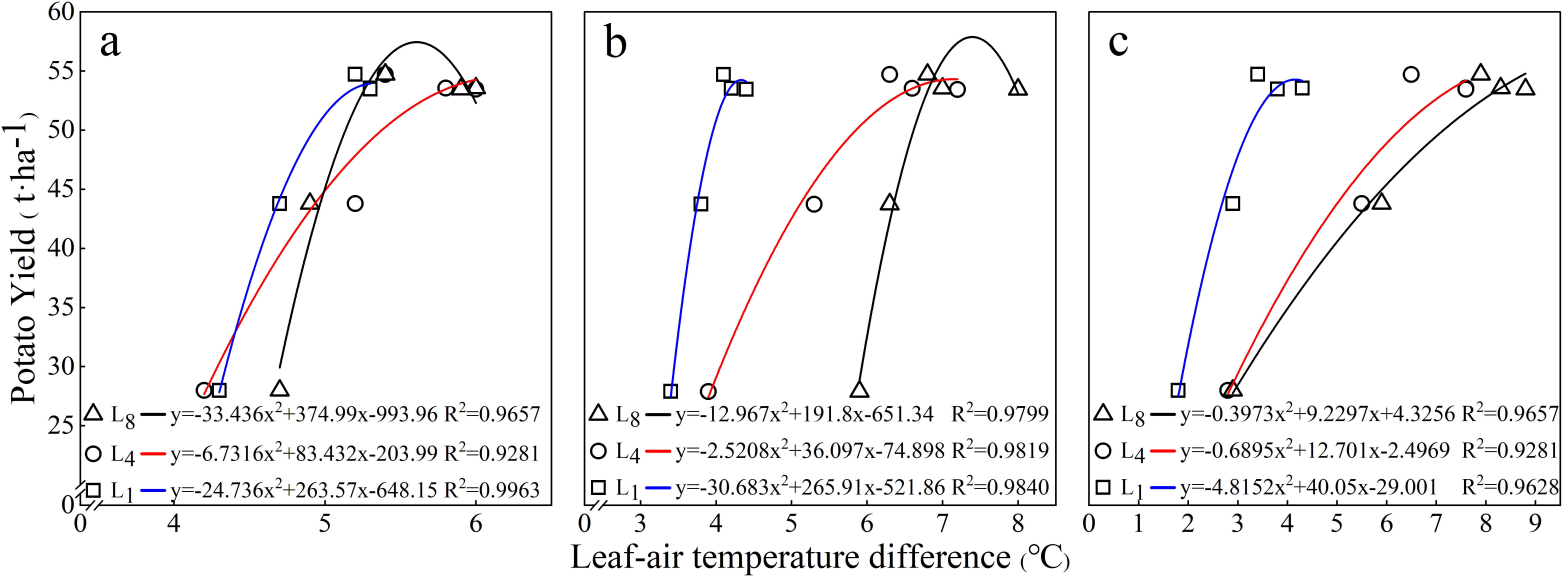
Regression analysis of the relationship between potato yield (y) and LAD (x) across different leaf positions and growth stages. **(a)** 35, **(b)** 50, and **(c)** 60 days after emergence. L_1_ is the top leaflet of the first compound leaf from the top of the plant. L_4_ and L_8_ are the top leaflets of the fourth and eighth compound leaves from the top, respectively.

Further statistical analysis showed that there was a significant binomial regression relationship (*p <* 0.05) between the potato yield and the LAD of each leaf at each growth stage, as shown in **Figure 3**. All of the regression equations clearly demonstrate that as the LAD increased, the potato yield also increased. However, once LAD reached a critical point, further increments in LAD no longer resulted in increased potato yield.

### 3.3 Relationships between LAD and plant water content

**Figure 4:**
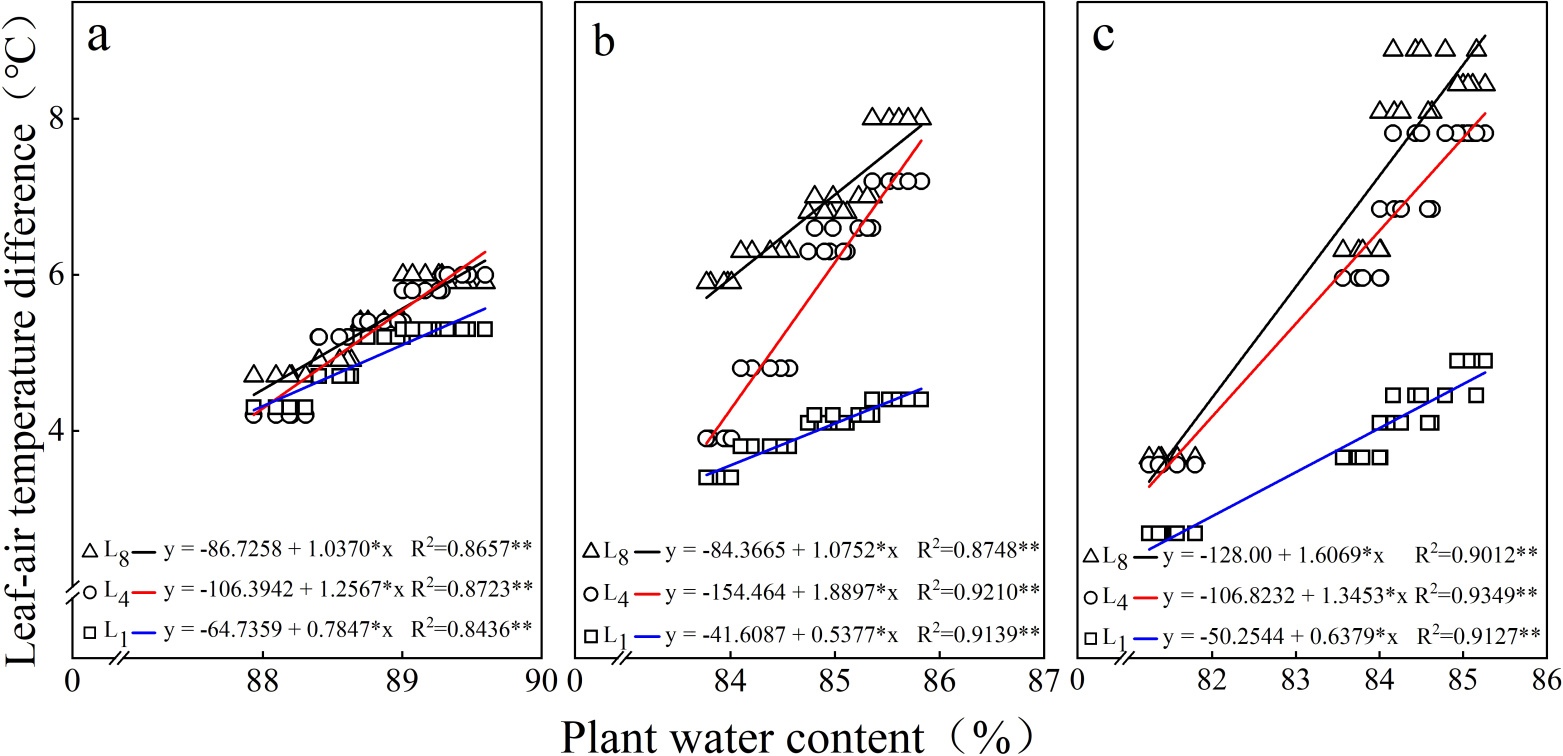
Relationships between the LAD and plant water content at **(a)** 35, **(b)** 50, and **(c)** 60 days after emergence. L_1_ is the top leaflet of the first compound leaf from the top of the plant. L_4_ and L_8_ are the top leaflets of the fourth and eighth compound leaves from the top, respectively.

There were significant linear relationships (*p* < 0.05) between the LAD of each leaf and the plant water content (PWC) across different growth stages. As the plant water content increased, the LAD exhibited diverse degrees of increase depending on leaf positions. At all growth stages, the coefficient of determination (*R* ^2^) of the regression for L_4_ was higher than that for other leaf positions (**Figure 4**).

Just as in the case of the relationship between LAD and PWC, a positive correlation (*p* < 0.05) between LAD and leaf relative water content (LRWC) was discerned at each growth stage. Throughout each growth stage, the correlation coefficients for L_4_ ranged from 0.48 to 0.59, being greater than those for L_1_ and L_8_ (**Table 6**).

**Table 6:** Correlations between leaf–air temperature (LAD) and leaf relative water content (LRWC) on different days after emergence.

Days after emergence (DAE)	Leaf position		
	L_1_	L_4_	L_8_
35	0.304**	0.480**	0.370**
50	0.364**	0.512**	0.323*
60	0.289*	0.594**	0.492**

L_1_ is the top leaflet of the first compound leaf from the top of the plant. L_4_ and L_8_ are the top leaflets of the fourth and eighth compound leaves from the top, respectively. Values are Pearson correlation coefficients. * and ** represent significance at *p <* 0.05 and *p* < 0.01, respectively.

### 3.4 Response of the LAD to soil water content

**Figure 5:**
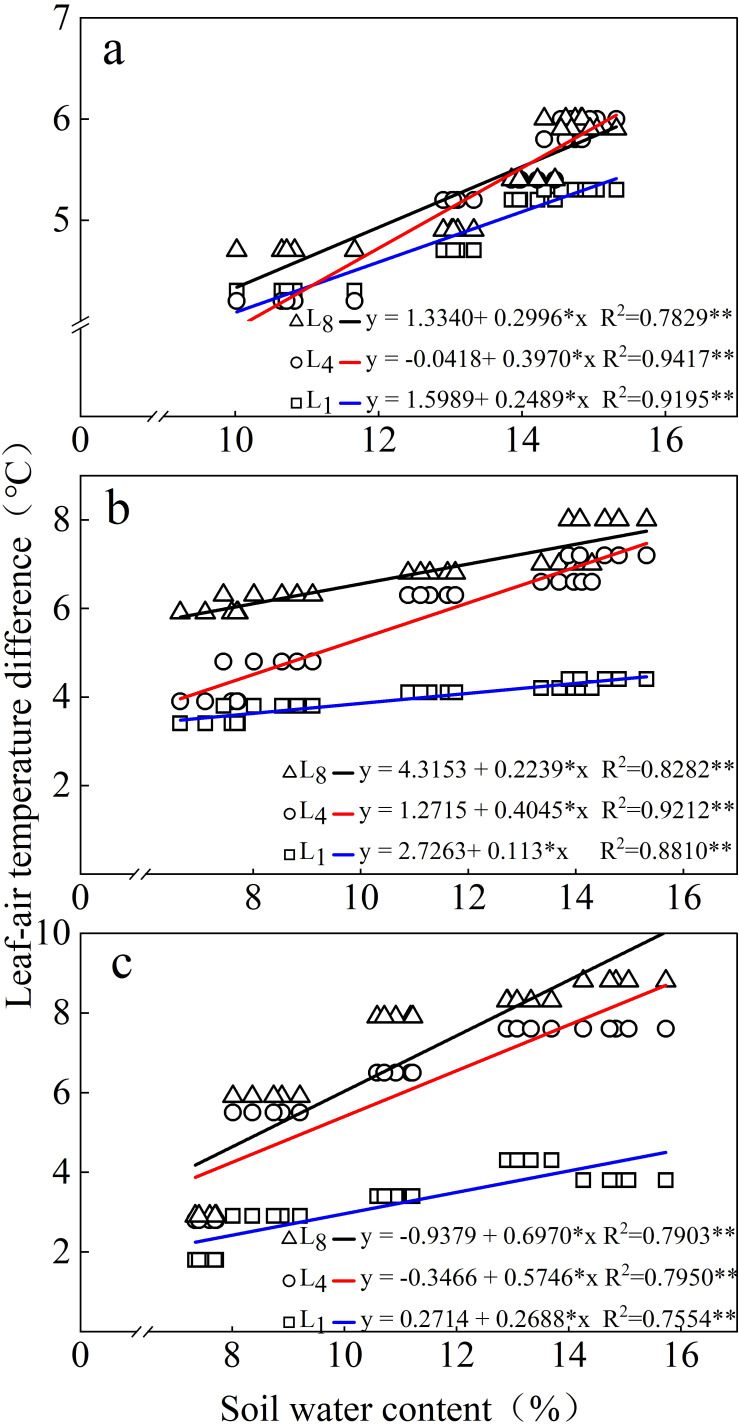
Relationships between the LAD and soil water content at **(a)** 35, **(b)** 50, and **(c)** 60 days after emergence in the 0–40-cm soil layer. L_1_ is the top leaflet of the first compound leaf from the top of the plant. L_4_ and L_8_ are the top leaflets of the fourth and eighth compound leaves from the top, respectively.

As the soil water content increased, the LAD increased to varying degrees depending on leaf positions—that is, the LADs of L_1_, L_4_, and L_8_, each to a different extent, reflected the soil water content status. For each leaf position, significant regression (*p* < 0.05) between LAD and soil water content was discerned. However, the *R* ^2^ of the regression for L_4_ was higher than that for other leaf positions at all growth stages, while the *R* ^2^ value for L_1_ was the lowest (**Figure 5**).

## 4 Discussion

### 4.1 Feasibility of assessing potato plant water status by measuring LAD

In this study, we systematically measured LAD at L_1_, L_4_, and L_8_ of potato plants across different growth stages and irrigation levels. The significant positive correlations between LAD and irrigation levels observed for each potato leaf position (**Tables 3**, **4**) clearly indicate that the LAD of potato responds highly to water supply. Notably, even the first leaf from the plant top (L_1_), which is typically incompletely expanded and exhibits substantial morphological variation among plants (Fan, 2019), demonstrated a significant positive correlation with irrigation level (**Table 4**). The relative water content of leaves (LRWC) is the best index to reflect the water balance of plants (Torres et al., 2019). In this study, the significant correlation observed between LAD and the corresponding leaf’s LRWC (**Table 6**) indicates that LAD could be an indicator of the water status in potato plants. The positive relationships between LAD and plant water content as well as between LAD and soil water content (**Figures 4**, **5**) validate that LAD can serve as a reliable indicator to monitor the water status of potato plants—that is, by measuring LAD, the water status of potato plants can be assessed.

The threshold LAD value, defined as the minimal LAD required to achieve maximum yield, is critical for developing LAD-based irrigation protocols. The yield saturation effect observed in the study (in which exceeding a critical LAD value no longer results in higher potato yields) and the significant binomial regression relationship between LAD and potato yield (*R* ^2^ > 0.92, *p* < 0.05) (**Figure 3**) provide two key operational advantages for precision irrigation in potato cultivation: (1) threshold-based irrigation triggering when LAD exceeds optimal ranges and (2) predictive yield modeling through continuous LAD monitoring and thereby further highlighting the substantial agronomic significance of monitoring LAD.

### 4.2 Optimal leaf position for assessing potato plant water status via LAD measurement

Previous studies have demonstrated that the leaf position has a significant impact on the water status diagnosis accuracy in gramineous crops through temperature measurement (Grant et al., 2016; Wang et al., 2023)—for instance, the LAD at two-thirds of the height of a maize plant could better reflect the moisture characteristics of the crop and soil (Wang et al., 2006), while in sorghum, the three leaves from the top of the plant were found most suitable for leaf temperature monitoring (Wang et al., 2019). They underscore the critical importance of leaf position selection in plant water status diagnostics. As potato has complex pinnately compound leaves and inherent intra-plant variability, precise leaf positioning becomes particularly essential for accurate water assessment. The results that the L_4_ exhibited significantly higher correlation coefficients between LAD and irrigation levels compared to L_1_ and L_8_ (**Table 4**) suggest a heightened sensitivity of L_4_ LAD to irrigation. Furthermore, the significantly higher *R* ^2^ values for L4 than for other leaf positions in modeling relationships between LAD and both PWC and SWC (**Figures 4**, **5**), along with the strongest correlations between LAD and LRWC (**Table 6**), collectively indicate L4’s superior responsiveness to dynamic plant water status changes. Notably, intra-treatment variability analysis shows that L_4_ maintained a significantly lower coefficient of variation (CV) in LAD measurements compared to L_1_ and L_8_ under identical irrigation regimes (**Table 5**). This is likely attributed to L_4_ being the first fully expanded mature leaf with stable morphological structures and physiological functions, whereas L_1_ represents developing tissue and L_8_ undergoes senescence. Considering its dual advantages of reduced variability and enhanced sensitivity to water availability, L_4_ can be proposed as the optimal leaf position for assessing potato plant water status via LAD measurement.

### 4.3 Potential future research directions

Potato plants have a relatively shallow and sparse root system, leading to inefficient water uptake capabilities (Zarzyńska et al., 2017; Zinta et al., 2022). This characteristic makes precision irrigation techniques particularly crucial for potato cultivation. The findings of this study lay the solid foundation for the development of precision irrigation management in potato production, being of substantial agronomic significance. However, implementing LAD measurements to guide irrigation in potato production relies on establishing appropriate LAD-based irrigation indices. Since the LAD-obtained relative maximum yield varies among years due to the variation of weather conditions, it probably needs several years of data to determine the threshold value of LAD of L_4_ for a certain potato production region. Moreover, this study revealed that as irrigation levels increased, the extent of the LAD increase varied with the growth stage. Specifically, the increase was more pronounced during the later growth stages (**Table 3**). This finding strongly suggests that LAD-based irrigation indices should be customized according to different potato growth stages.

## 5 Conclusions

This study demonstrates that the LAD is a reliable indicator for monitoring the water status of potato plants, and the L4 (4th leaf from the apex) is identified as the optimal leaf position for assessing the water status due to:

1. Enhanced sensitivity: L_4_-LAD showed the highest responsiveness to irrigation gradients and dynamic change in plant water contents;
2. Reduced variability: Under uniform irrigation, L_4_-LAD exhibited significantly lower variability among individual plants.

In addition, a significant binomial regression relationship is identified between potato yield and LAD at each leaf position across all growth stages, showing a yield saturation effect in which exceeding a critical LAD value no longer results in higher potato yields. This provides threshold-based triggering of irrigation when LAD exceeds optimal ranges. Overall, the study lays a foundation for precision irrigation in potato production, enabling improved water use efficiency and sustainable potato production in a semi-arid region.

## Data Availability

The original contributions presented in the study are included in the article/supplementary material. Further inquiries can be directed to the corresponding authors.
